# Quality care outcomes following transitional care interventions for older people from hospital to home: a systematic review

**DOI:** 10.1186/1472-6963-14-346

**Published:** 2014-08-15

**Authors:** Jacqueline Allen, Alison M Hutchinson, Rhonda Brown, Patricia M Livingston

**Affiliations:** Deakin University, School of Nursing and Midwifery, 221 Burwood Hwy, Burwood, 3125 Vic Australia; Deakin University, School of Nursing and Midwifery; Centre for Nursing Research – Deakin University and Monash Health Partnership, Monash Health, 221 Burwood Hwy, Burwood, 3125 Vic Australia; Faculty of Health & School of Nursing and Midwifery, Deakin University, 221 Burwood Hwy, Burwood, 3125 Vic Australia

**Keywords:** Transitional care, Discharge care, Discharge planning, Older person care, Aging, Systematic review

## Abstract

**Background:**

Provision of high quality transitional care is a challenge for health care providers in many western countries. This systematic review was conducted to (1) identify and synthesise research, using randomised control trial designs, on the quality of transitional care interventions compared with standard hospital discharge for older people with chronic illnesses, and (2) make recommendations for research and practice.

**Methods:**

Eight databases were searched; CINAHL, Psychinfo, Medline, Proquest, Academic Search Complete, Masterfile Premier, SocIndex, Humanities and Social Sciences Collection, in addition to the Cochrane Collaboration, Joanna Briggs Institute and Google Scholar. Results were screened to identify peer reviewed journal articles reporting analysis of quality indicator outcomes in relation to a transitional care intervention involving discharge care in hospital and follow-up support in the home. Studies were limited to those published between January 1990 and May 2013. Study participants included people 60 years of age or older living in their own homes who were undergoing care transitions from hospital to home. Data relating to study characteristics and research findings were extracted from the included articles. Two reviewers independently assessed studies for risk of bias.

**Results:**

Twelve articles met the inclusion criteria. Transitional care interventions reported in most studies reduced re-hospitalizations, with the exception of general practitioner and primary care nurse models. All 12 studies included outcome measures of re-hospitalization and length of stay indicating a quality focus on effectiveness, efficiency, and safety/risk. Patient satisfaction was assessed in six of the 12 studies and was mostly found to be high. Other outcomes reflecting person and family centred care were limited including those pertaining to the patient and carer experience, carer burden and support, and emotional support for older people and their carers. Limited outcome measures were reported reflecting timeliness, equity, efficiencies for community providers, and symptom management.

**Conclusions:**

Gaps in the evidence base were apparent in the quality domains of timeliness, equity, efficiencies for community providers, effectiveness/symptom management, and domains of person and family centred care. Further research that involves the person and their family/caregiver in transitional care interventions is needed.

**Electronic supplementary material:**

The online version of this article (doi:10.1186/1472-6963-14-346) contains supplementary material, which is available to authorized users.

## Background

Older people with complex comorbid health problems are frequently required to transition between hospital and home during an episode of acute illness. Estimates from the United States suggest that at least 20% of older Medicare recipients with five or more chronic conditions require frequent inpatient and emergency care from hospitals [1, 2]. The provision of high quality transitional care continues to be a challenge in many western countries because of a continued focus on acute, episodic care [3–7]. In Western countries, health care quality standards and expectations emphasise effective, efficient, safe, timely and equitable care in addition to person and family centred care [4, 6–8]. However, previous researchers have found that studies investigating transitional care interventions have focussed on re-hospitalization rates and cost containment for inpatient providers [9–12]. This suggests that other indicators of quality of care have received less attention. These indicators include other domains of care effectiveness, efficiency and safety: for example; symptom management, self-management and efficiencies for community providers. Additionally, quality indicators of timeliness, equity and person and family centred care have received limited focus in research outcomes. This review was conducted to synthesise the evidence in relation to quality outcomes following transitional care for older people and their caregivers transferring from hospital to home in order to make recommendations for research and practice.

### Demand on health services

The frequency and complexity of care transitions for older people is expected to increase considerably along with the predicted increases in demand on health and aged care services [13]. Globally, populations are aging due to declines in fertility and increases in life expectancy [14–16]. One effect of the aging population is the growing numbers of older people living with chronic illness who are expected to require extensive health and aged care from multiple providers and across multiple care settings [13, 17, 18]. Some policy makers and health planners in Australia have predicted that the numbers of people in the future workforce will be unable to sustain the resources required to support older people [5, 13]. There is a risk, however, that these views may be used to justify suboptimal health and aged care for older people [19]. Importantly, societies can adapt to changes in population aging and adopt age inclusive policies and practices [16, 19].

Quality in health care standards and indicators recommended in the United States of America [4], United Kingdom [8] and Australia [6] (see Table 1) include: effectiveness, efficiency, safety and risk, timeliness, equity and person and family centred care, offer opportunities and guidance for optimal health and aged care for older people including optimal transitional care from hospital to home. Research in the field of transitional care that is focussed on quality indicators is vital in guiding age centred policies and practices.

**Table 1:** **Definition of quality indicators**

Quality indicator	Definition of indicator
Effectiveness	Effective care is based in evidence and is provided to the people most likely to benefit [4, 6, 8].
Efficiency	Efficient care is care without waste, including wasted resources [4].
Timeliness	Timely care is care that is provided in a timely manner without lengthy waiting periods for patients and their family [4].
Safety and risk	Care is low risk and safe when it causes no harm to patients, families or health care staff [4, 6, 8].
Equity	Care that is fair to everyone. No group of people receive inferior care based in differences in gender, culture, ethnicity, age, sexuality, geographic location or socioeconomic status [4].
Person and family centred care and experience	Care that is respectful of patients and families preferences, values and goals. Care decisions involve patients and families [4, 6, 8].

### Transitional care

Transitional care is a broad term for care interventions that promote safe and timely transfer of patients between levels of care and across care settings [20–23]. Transitional care is not strictly defined by beginning and end points; it includes pre hospital discharge activities and immediate post hospital discharge follow-up at the next location of care [21, 24]. Transitional care can be considered a part of integrated care, which occurs over longer duration of care episodes [25] and it can be considered as a part of prevention of re-hospitalization programs within longer-term chronic disease management initiatives [26]. Although transitional care is related to integrated care and prevention of re-hospitalization programs, it is considered a conceptually distinct category of care interventions [24]. According to Coleman and Boult [20], there are a number of essential elements in quality transitional care: communication between providers about the discharge assessment and plan of care, preparation of the patient and carer for the care transition, reconciliation of medications at transition, a plan for follow-up, and patient education about self-management.

Preventable adverse events, including medication errors, falls, errors in diagnosis, post-operative infections and confused states, are risks for older people during care transitions, particularly those with functional difficulty and chronic illness [11, 27]. Consequently, discharge planning that occurs solely within the acute inpatient setting without in-home follow-up support is not sufficient in the case of many older people with chronic illnesses and functional difficulty [22, 25, 28]. The success of care transitions for many older people depends on holistic transitional care interventions involving both hospital discharge planning and in-home follow-up and support [13, 29].

### What is currently known about quality transitions?

Reviews of the literature in transitional care interventions have focussed on assessing outcomes of re-hospitalization and length of stay with mixed findings [24, 27, 30–33]. Although in a recent Cochrane systematic review of transitional care nested within disease focussed models, Shepperd and colleagues [12] concluded that transitional care was “probably” effective in reducing rates of re-hospitalization in the immediate post discharge period and reducing length of stay. Shepperd and associates [12] further concluded that cost was most likely shifted from the inpatient to the community sector.

Risk has also been a focus in the literature. Mansah and colleagues [27] found that pharmacist led interventions in medication reconciliation reduced adverse events associated with non-adherence with medications in the home. Laugaland and colleagues [11] further identified transitional care interventions that reduced adverse events post discharge as those that commenced early in hospitalisation, involved key workers/discharge coordinators, included patients and family carers, involved a multidisciplinary and multicomponent approach, and reconciled medications. Some reviewers nominated re-hospitalization rates as an outcome capturing risk and safety following transitional care [27, 30, 34]. Effective communication between health providers during care transitions of older people has also been identified as important in reducing risks and adverse outcomes [11, 27, 34, 35].

Numerous reviewers have identified limited research and mixed findings about person and family centred experiences during care transitions and outcomes following transitional care interventions [10, 12, 24, 31, 32, 35–37]. These findings indicate that the older person’s experience and the experiences of their family/carer have not received sufficient attention in the transitional care intervention research to date.

Transitional care for older people has been evaluated largely in terms of re-hospitalization rates, thereby capturing specific dimensions of quality such as effectiveness, efficiency for inpatient providers, and risk and safety [27, 30]. Other important dimensions of quality in health care; person and family centred care, symptom management, efficiencies for community providers, timeliness and equity, have not received the same focus. Additionally, a research emphasis on reducing rates of re-hospitalization may unintentionally and subtly contribute to the exclusion of older people from health care [5, 13, 16, 19]. A holistic understanding of quality of care transitions is therefore required if transitional care providers and researchers are to assist societies and health care systems to adapt positively to changes associated with population aging.

### Objectives

This systematic review was conducted to:

1. Locate and synthesise research using randomised control trial designs on quality of outcomes following transitional care interventions compared with standard hospital discharge for older people with chronic illnesses.
2. Make recommendations for research and practice.

## Methods

This systematic review synthesised published studies, using randomised controlled trial designs, to investigate the effects of transitional care interventions for people aged 60 years and older on health care outcomes. Cochrane Collaboration guidelines [38] were used to direct the review.

### Search strategy

A search for peer-reviewed journal articles was conducted using the search terms: 'discharge planning’, ' hospital discharge’, 'discharge care pathways’, 'discharge care protocols’, 'transitional care’, 'transitional care pathways’, 'transitional care protocols’. These terms were added to the phrase 'from the inpatient setting to the home’ to form concept groups. These concept groups were further combined with 'aged care’ and similar terms (aging, geriatrics, gerontology and older person care), and 'community’ and similar terms (home care, primary care, domiciliary care). Eight databases were searched: CINAHL, Psychinfo, Medline, Proquest, Academic Search Complete, Masterfile Premier, SocIndex, Humanities and Social Sciences Collection, in addition to the Cochrane Collaboration, Joanna Briggs Institute and Google Scholar.

Studies were limited to those published between January 1990 and May 2013. This timeframe was chosen due to the development since the 1990s in many western countries of community-based care programs and the evolution of more formally structured transitional care interventions inclusive of discharge processes and in-home follow-up [21, 39, 40].

### Inclusion criteria

To be included, an article was required to (1) be published in a peer reviewed journal, (2) report on a transitional care intervention compared with standard hospital discharge, (3) use a randomized control trial design, (4) be published in English, and (5) provide an analysis of outcomes that evaluated quality indicators related to older people. Transitional care included any intervention applied in the inpatient setting, inclusive of follow-up in the community. All studies included people 60 years of age or older. Sixty years of age was selected because it was the definition of 'older adult’ used by the World Health Organization [13, 41].

### Screening procedure

Articles were entered into an Endnote version 16 database [42] for screening and duplicates were removed. Two reviewers independently screened the title and abstract of each study to identify articles meeting the inclusion criteria. Records for which relevance could not be determined based on title and abstract alone were screened from the full text journal article. Discrepancies in reviewers’ decisions regarding relevance for inclusion were resolved by consensus.

### Data extraction

Additionally, a data extraction tool was devised, based on an earlier literature review [9], to capture the main features of studies meeting the inclusion criteria (see Additional file 1). A single reviewer extracted the data for all included studies. Due to the heterogeneity in the transitional care interventions and outcomes, data were presented in tables and were not pooled.

### Assessment for risk of bias

The Cochrane Collaboration’s tool for the assessment of bias in randomized controlled trials was used to assess for bias in all included studies [38]. This tool appraises numerous areas of potential bias; selection bias, performance bias, detection bias, attrition bias, reporting bias and 'other’ sources of bias. The potential for selection bias is assessed in terms of the adequacy of randomisation processes (random sequence generation) and the adequacy of the concealment of allocation to intervention group (allocation concealment) [38]. Performance bias is the study bias that may result from the knowledge of research participants and research staff of the interventions that participants were allocated to. Detection bias is possible when outcome assessors know which interventions participants were allocated to [38]. Attrition bias is the potential for biased conclusions resulting from incomplete outcome data. Reporting bias may result from the selection of particular outcomes for reporting [38]. The potential for other sources of bias (other bias) was also appraised. Two reviewers independently assessed included studies for study bias in accordance with the guidelines for the bias assessment tool [38]. Both reviewers then met to compare their findings. Consistency rates between reviewers were high (>80%) with minor discrepancies resolved through discussion with a third reviewer.

## Results

**Figure 1:**
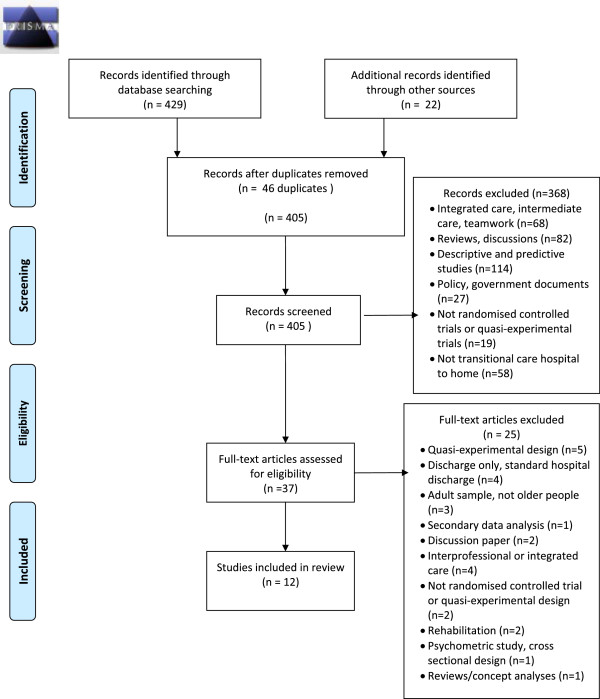
**Literature review search.**

The search identified 405 records. Of these, 12 published journal articles met the inclusion criteria. Outcomes from the search and screening results are presented in Figure 1.

Title and abstract screening resulted in the exclusion of 368 articles. Full texts for 37 articles were retrieved and a further 25 articles were excluded. Reasons for the exclusion of these articles are presented in Table 2.

**Table 2:** **Reason for exclusion for studies retrieved in full text (n = 25)**

First author (year)	Reason for exclusion
Arbaje (2010) [43]	Quasi-experimental design
Bull (2000) [44]	Not a full transitional care intervention, discharge planning
Balaban (2008) [45]	Adult sample, not focussed on older people with functional difficulty
Bonnet-Zamponi (2013) [46]	Secondary data analysis, drug related problems
Brand (2004) [47]	Quasi-experimental design
Coleman (2004) [48]	Quasi-experimental design
Dedhia (2009) [49]	Quasi-experimental design
Einstadter (1996) [50]	Not a full transitional care intervention, discharge planning
Golden (2010) [51]	Not a full transitional care intervention, discussion paper
Haggmark (1997) [52]	Not a full transitional care intervention, interprofessional care
Ham (2011) [3]	Not a full transitional care intervention, integrated care
Hansen (1992) [53]	Not a full transitional care intervention
Hebert (2008a) [54]	Not a full transitional care intervention, integrated care
Hebert (2008b) [55]	Not a full transitional care intervention, integrated care
Hegney (2002) [56]	Not a full transitional care intervention, discharge planning
Jack (2009) [57]	Adult sample, not focussed on older people with functional difficulty
Jeangsawang (2012) [58]	Not randomised controlled trial or quasi-experimental design
Lattimer (2012) [59]	Discussion paper
Melton (2012) [60]	Adult sample, not focussed on older people with functional difficulty
O’Reilly (2008) [61]	Rehabilitation, not transitional care
Ornstein (2010) [62]	Mixed methods, not randomised controlled trial, not quasi-experimental design
Parker (2004) [30]	Systematic review
Parker (2009) [63]	Rehabilitation, not transitional care
Parry (2008) [64]	Psychometric study, cross sectional design
Steeman (2006) [65]	Quasi-experimental design

In total 5,269 older people were included across 12 randomised controlled trial studies conducted in four western countries: USA (7 studies), Australia (3 studies), Denmark (1 study), and France (1 study).

### Transitional care interventions

Each of the transitional care interventions tested in the 12 studies [66–77] contained elements considered essential to high quality transitional care: discharge assessment and care planning, communication between providers, preparation of the person and carer for the care transition, reconciliation of medications at transition, community-based follow-up, and patient education about self-management [20, 24]. The main practitioner/s responsible for implementing the transitional care intervention varied across the 12 studies. Advanced practice nurses (nurses educationally prepared at Masters degree level) implemented the transitional care in five studies [68, 72, 74, 75, 77]. General practitioners (physicians in primary care) and primary care nurses (nurses educationally prepared at either Bachelor degree level or diploma level in primary care, also referred to as practice nurses) implemented transitional care in three studies [66, 67, 73]. The older person and their carer implemented their own transitional care with the support of a transition coach in the study by Coleman et al. [69]. Case managers were responsible for care transitions in the study by Lim et al. [76] and geriatricians were responsible for transitional care in the studies by Hansen et al. [70] and Legrain et al. [71].

With the exception of the self-management and transition coaching intervention described by Coleman et al. [69] there was limited reporting on the involvement of older people and their carers/family in the development of the transitional care intervention. Coleman et al. [69] reported that their intervention was informed by focus groups with older people and their families/carers who articulated what was important to them in quality care transitions and what sort of assistance they wanted in these care episodes [64].

The main limitations identified across the 12 studies were in relation to the generalizability of findings. Findings would only be generalizable to those people with similar characteristics to those included in the sample and to the practitioners implementing the intervention.

Characteristics of the included studies are presented in Table 3.

**Table 3:** **Study characteristics - included studies (n = 12)**

First author (year)	Setting, sample & study design	Stated aims	Intervention	Limitations
*Discharge protocol & advanced practice nurse*				
Naylor (1990) [72]	US, acute inpatient (medical, surgical) to home	To test a protocol of discharge planning compared with standard hospital discharge	Protocol implemented by advanced practice nurses (APN):	Small sample size
				Costs of nursing intervention was incomplete due to missing data
	N = 40, average age 78.8 years			
			Assessment and discharge planning within 24 hours of admission	
	Chronic illness		Discharge plan included health teaching to be conducted in primary care	
	RCT			
			APN telephone follow up for 2 weeks post discharge	
Naylor (1994) [68]	US, acute inpatient (medical, surgical) to home	To assess an APN implemented discharge planning protocol compared with standard hospital discharge	Discharge planning protocol implemented by APNs:	Generalizability of findings is limited to older people with cardiovascular diagnoses, oriented and alert at admission, well educated, with good support systems and few functional deficits
			Discharge assessment 24–48 hours after hospital admission	
			Discharge plan developed collaboratively with client, carer, multidisciplinary team	
	N = 276, average age 76 years			
			Communication and coordination maintained by APN throughout this process with multidisciplinary team including primary care providers	
	Chronic illness			
	RCT			
			Post discharge APN phone availability	
Naylor (1999) [77]	US, acute inpatient (medical, surgical) to home	To assess an APN implemented discharge planning protocol compared with standard hospital discharge for older people at risk of re-hospitalization	APN protocol discharge planning and home support follow up:	Generalizability of findings is limited to older people oriented and alert at admission
			APN care continuity	
				Intervention may be limited to deployment by advanced practice nurses in primary care
			APN conducted hospital discharge planning care and in home support (substituted for the visiting nurse) for the first 4 weeks post discharge	
	N = 363, average age 75.4 years			
			APN individualised patient care in collaboration with the person’s physician	
	Chronic illnesses			
	RCT			
Naylor (2004) [75]	US, acute care to home	To assess the effects of an advanced practice nurse delivered transitional care intervention on older people with heart failure and comorbid conditions	Advanced practice nurse conducted a transitional care intervention emphasising	Generalizability of findings is limited to older people with exacerbation of cardiac failure and co morbid conditions
	N = 239, average age 76 years		Discharge assessment	
				Intervention may be limited to deployment by advanced practice nurses
			Discharge plan	
			Discharge coordination with multidisciplinary team	
			APN care continuity	
			Education	
	Heart failure and comorbid illnesses		Symptom management and self-management	
			Goal setting	
			Medication management	
			Home visits/home nursing up to 3 months following discharge	
	RCT			
Enguidanos (2012) [74]	US, acute care to home	To assess impact of brief nurse practitioner (NP) intervention for older people discharged from hospital to home compared with standard hospital discharge	NP in primary care conducted:	Sample size insufficiently powered to detect an effect of the intervention
			Education about discharge instructions to older person	
				Intervention may be limited to deployment by nurse practitioners
	N = 199, average age 73.58 years		Medication reconciliation	
			Home care needs assessment and referral to resources	
			Scheduling follow-up medical appointments	
	Chronic illnesses			
	RCT			
*General practitioner and primary care nurse models*				
Weinberger (1996) [67]	US, acute care to home	To test an primary care intervention on rates of re-hospitalization, length of stay, quality of life and veteran satisfaction compared with standard discharge care	Before discharge:	Generalizability of study limited to older US male veterans
			The primary care nurse conducted the discharge assessment, provided education and the contact telephone numbers of the primary care nurse and general practitioner (GP), and scheduled an appointment within 2 days of discharge to attend the primary care clinic	Substantial primary care resources were required to implement the intervention
	N = 1396, average age 63 years, veteran sample, mostly male (98.5%)			
			The GP visited the veteran in hospital within 2 days prior to discharge and reviewed the discharge plan, medication, and medical problems with the hospital physicians	
	Chronic illnesses		After discharge:	
			The primary care nurse telephoned the patient (within 2 working days of discharge) at home to assess any difficulties with medications/medical treatments, health problems, remind of follow-up appointment	
			Patients were followed-up in clinic	
			The primary care nurse and GP reviewed treatment plan at first appointment.	
	RCT			
McInnes (1999) [73]	Australia, acute hospital-geriatric care unit (patients admitted under care of geriatrician) to home	To test if GP involvement in discharge planning patient outcomes when compared with standard hospital discharge	Standard hospital discharge practice with the addition of GP visit pre discharge:	Of those randomized to the intervention group only 52% of patients were actually visited by their GP in hospital
			GPs invited to undertake pre discharge visit:	
	N = 364, average age 81 years			Substantial primary care resources were required
			Information sought from GP re recommendations for post discharge care	
	RCT			
			GP able to discuss care/treatment with hospital based medical and allied	
			health staff	
			GP had access to the patient’s hospital care record during the visit	
Preen (2005) [66]	Australia, acute hospital to home	To test a hospital coordinated discharge plan that involved the GP when compared with standard hospital discharge	Research nurse based in the hospital:	Intervention was not fully implemented as only 42% of GPs returned the discharge plan to the hospital prior to discharge
			Developed discharge plan (determined client discharge problems, goals and community service provider involvement)	
				Sample size may have been insufficiently powered to detect an effect of the intervention
			Faxed the discharge plan to the GP 24–48 hours prior to discharge	
	N = 189, average age 75 years		The GP	
			Reviewed the discharge plan, modified it and returned it to the hospital by fax	
	Chronic illnesses			
			Research nurse based in the hospital:	
			Explained the discharge plan to the client	
	RCT		Provided copies of the discharge plan to the client, and all service providers identified on the care plan.	
			Scheduled an appointment with the GP	
*Self-management and transition coaching*				
Coleman (2006) [69]	US, acute hospital to home	To assess the effects of a care transitions intervention in comparison with standard hospital discharge care, using RCT design, on rehospitalisation rates for older people	Care Transitions Intervention (as per Coleman et al. 2004 above) Intervention developed from qualitative research with older people and their care givers about what would be most valuable to them during care transitions:	Intervention may be limited to deployment by advanced practice nurses in the role of transition coach
	N = 747, average age 76 years			
			Medication assistance and self-management	
	Chronic illnesses		Patient centred and owned record	
			Timely follow-up from primary care providers	
	RCT		List of problem triggers indicating deterioration in their particular chronic illness and what to do about these	
*Discharge case management*				
Lim (2003) [76]	Australia, acute hospital to home	To test the effects of case management and post acute care services on organisation and patient/caregiver outcomes in comparison with standard hospital discharge	Post Acute Care program:	Costs were averages of community services and daily hospital bed utilisation rates, actual costs for each individual were not captured
			Short term case management and provision of post-acute care services (in home) nursing, allied health, community supports	
	N = 598, average age 76 years			
	Chronic illnesses			
	RCT			
*Inpatient geriatric evaluation, co-management (with ward staff) and transitional care*				
Hansen (1995) [70]	Denmark, subacute geriatric ward to home	To compare the intervention with standard hospital discharge on the number of medical and social problems after discharge, the need for modification of the discharge plan after discharge and rates of re-hospitalization to hospital	The Geriatric Evaluation and Management team (geriatrician, nurse and physical therapist) supported inpatient discharge planning and follow-up at home	Generalizability of findings limited to older people with low functioning
	N = 193, average age intervention 78 to 80 years			Intervention may be limited to deployment by geriatricians
			Follow-up involved re-evaluation and modification of the care plan, communication with the primary care team (GP, community nurse) during home visits at 1, 3, 8, 16 weeks following discharge	
	Multiple chronic conditions and low functional status			
	RCT			
Legrain (2011) [71]	France, acute inpatient geriatric care unit to varying locations: home, nursing home, rehabilitation unit, acute care unit	To compare a comprehensive discharge intervention with standard hospital discharge on emergency department visits and re-hospitalisations	Geriatrician delivered inpatient intervention:	Findings generalizable to functionally dependent older people
			Medication review	
			Education re self-management of disease	
			Communication principally with GP	Intervention may be limited to deployment by geriatricians
			Screening for main risks for frail elderly	
			Depression	
	N = 665, average age 86 years			
	Chronic illnesses			
	RCT			

### Outcomes

Table 4 presents a summary of the outcome findings of the randomised controlled trial studies. Table 5 presents a summary of quality indicators measured in randomised controlled trial study outcomes.

**Table 4:** **Main findings - included studies (n = 12)**

First author (year)	Main findings
*Discharge protocol & advanced practice nurse*	
Naylor (1990) [72]	Significant reduction in rates of re-hospitalization for intervention group over the 12 weeks post discharge
	No difference in length of stay
	No difference in posthospital infections
Naylor (1994) [68]	Intervention patients in the medical units at 6 week follow-up experienced:
	Significant delay in re-hospitalization to hospital
	Fewer total days of re-hospitalisation
	Lower health care costs (inclusive of inpatient, clinic, home visits)
	No change in functional status, mental status, self-esteem or affect
	Intervention caregivers up to 12 weeks following discharge experienced:
	No change in functional status, caregiving demands, family
	functioning, affect
Naylor (1999) [77]	Intervention group at 24 week follow-up experienced fewer:
	Re-hospitalizations
	Hospital days per patient
	Lower costs than control group
	No statistically significant differences in functional status, depression or patient satisfaction between groups
Naylor (2004) [75]	The time to first admission was longer in intervention patients
	At 52 weeks, intervention patients had fewer re-hospitalizations and lower total mean costs
	There were short term improvements among intervention patients in quality of life (physical domain, up to 12 weeks post discharge) and satisfaction with discharge and transition care (up to 6 weeks post discharge)
Enguidanos (2012) [74]	No change in re-hospitalization rates at 6 months following enrolment in the study
	The intervention group experienced significantly fewer visits to GPs
	There were no changes between intervention and control groups in self-efficacy or satisfaction with service
*General practitioner and primary care nurse models*	
Weinberger (1996) [67]	At 6 months following discharge:
	Intervention group had significantly higher rates of re-hospitalization and if re-admitted longer in hospital stay than controls (discharge as usual).
	Intervention group were significantly more satisfied with their care than controls
	No differences in quality of life scores between groups
	Quality of life scores were low in both groups
McInnes (1999) [73]	At 6, 12, 26 weeks following discharge:
	No significant differences in length of stay, rates of re-hospitalization or time to first re-hospitalization
	Intervention patients were significantly more likely to be
	Referred to community services at discharge and report that hospital staff had discussed their discharge plan with them
	Intervention patients reported increased satisfaction with discharge arrangements and preparation
Preen (2005) [66]	There were no differences in length of stay between groups
	One week following discharge:
	GPs in the intervention group were more satisfied with the documentation
	Discharge communication to GPs in the intervention was significantly faster than for GPs in the control group
	Patients in the intervention group reported improved satisfaction with discharge planning, access to health services, confidence with discharge, and mental quality of life
*Self-management and transition coaching*	
Coleman (2006) [69]	Intervention group had significantly lower re-hospitalization rates than the control group at 30, 90 and 180 days post discharge
	Intervention group had significantly lower hospital costs than the control group at 30, 90 and 180 days post discharge
*Discharge case management*	
Lim (2003) [76]	Over 6 month follow-up period there were no differences in rates of unplanned re-hospitalizations
	Intervention patients had significantly reduced length of stay (index hospitalisation)
	Costs (hospital utilisation) lower in intervention patients over 6
	months following discharge
	No differences in costs (utilisation of community services)
	between groups
	Significantly improved self-reported quality of life in intervention patients at one month follow-up
	No difference in caregiver burden at 1 month follow-up
*Inpatient geriatric evaluation, co-management (with ward staff) and transitional care*	
Hansen (1995) [70]	At 6 months following discharge:
	People in the intervention group were significantly less likely to be re-admitted to hospital than those in the control group
	There were no differences in rates of admission to nursing homes or mortality rates
	Significant increase in new and unforseen problems identified following discharge in people receiving the intervention.
	Intervention participants were significantly more likely to be allocated home help.
Legrain (2011) [71]	Older people in the intervention group were significantly less likely to attend the emergency department or be re-admitted at 3 months following discharge
	There were no differences between groups in ED attendances or re-hospitalizations at 6 months following discharge

**Table 5:** **Quality indicators assessed in study outcomes- included studies (n = 12)**

First author (year)	Quality indicators assessed in study outcomes					
	Effectiveness	Efficiency	Timeliness	Safety & risk	Equity	Person & family centred care
• *Discharge protocol & advanced practice nurse*						
Naylor (1990) [72]	✔	✔		✔		
Naylor (1994) [68]	✔	✔		✔		
Naylor (1999) [77]	✔	✔		✔		✔
Naylor (2004) [75]	✔	✔		✔		✔
Enguidanos (2012) [74]	✔	✔		✔		✔
• *General practitioner and primary care nurse models*						
Weinberger (1996) [67]	✔	✔		✔		✔
McInnes (1999) [73]	✔	✔		✔	✔	✔
Preen (2005) [66]	✔	✔	✔	✔	✔	✔
• *Self-management and transitional coaching*						
Coleman (2006) [69]	✔	✔		✔		
• *Discharge case management*						
Lim (2003) [76]	✔	✔		✔		
• *Inpatient geriatric evaluation, co-management and transitional care*						
Hansen (1995) [70]	✔	✔		✔	✔	
Legrain (2011) [71]	✔	✔		✔		

#### Efficiency, effectiveness and safety

Re-hospitalization rates, length of stay, and costs are considered important indicators of efficiency, effectiveness and patient safety [4, 6]. Eleven of the twelve studies measured re-hospitalization rates following the transitional care intervention [67–77], and three studies measured length of stay [66, 73], [86]. In six studies, significant reductions in re-hospitalization rates were found for people in the intervention groups at up to six months following hospital discharge [68–70, 72, 75, 77] and at up to three months following discharge in the study by Legrain et al. [71]. Three studies did not find any difference in re-hospitalization rates between treatment and control groups at up to six month follow up [73, 74, 76]. One study by Weinberger et al. [67] found the veterans in the intervention group had significantly higher rates of re-hospitalization than veterans in the control group. Weinberger et al. [67] speculated that the veterans in their study were experiencing very poor health and that the transitional care intervention assisted in early identification of health difficulties requiring re-hospitalisation.

Lim et al. [76] found reduced length of stay when older people were re-admitted following the intervention. Two studies [66, 73] found no significant differences in length of stay between intervention and control groups. One study by Weinberger et al. [67] found the veterans in the intervention group who were re-admitted had a longer stay in hospital than veterans in the control group.

Two studies assessing the effectiveness of general practitioner and practice nurse interventions on re-hospitalization rates [67, 73] or on length of stay [66, 73] did not find significant improvements in these outcomes.

Costs were assessed in three studies [68, 75, 77]. In each of these studies [68, 75, 77], costs were reduced for those people who received the intervention. Efficiencies for community providers were assessed in only one study. Enguidanos et al. [74] found fewer visits to general practitioners were required for those people who received the intervention.

Other quality indicators were assessed to determine the effectiveness of transitional care. Of the 12 included studies, two studies addressed functional status (Naylor et al. [77] and Naylor et al. [75]). Neither study found statistically significant differences on these measures for people who received the intervention. The study by Naylor et al. [77] assessed depressive symptoms following the intervention and found no statistically significant differences between intervention and control groups. Although assessment of re-hospitalization rates is inclusive of symptom control, few studies specifically measured symptom management following discharge or transitional care.

Quality of life was assessed in four studies [66, 67, 75, 76]. Naylor et al. [75] found an improvement for people who had participated in the intervention in physical quality of life. Preen et al. [66] found a significant improvement in mental quality of life for people who received the intervention one week following discharge. Lim et al. [76] found quality of life was better in people who had participated in the intervention at one-month follow-up. Weinberger et al. [67] found no differences in quality of life scores between veterans in their intervention and control groups.

#### Person and family centred care

Person and family centred care is considered essential to the quality of health care provision [4]. Patient satisfaction was measured in six of the 12 identified studies [66, 67, 73–75, 77]. Naylor et al. [75], Weinberger et al. [67], McInnes et al. [73], and Preen et al. [66] found that patient satisfaction scores for older people in the intervention groups were significantly improved compared with standard hospital discharge. Naylor et al. [77] and Enguidanos et al. [74] found no improvements in patient satisfaction following implementation of the transitional care intervention. Caregiver burden was measured in two studies [68, 76]. Naylor et al. [68] and Lim et al. [76] found no change in caregiver burden at one-month follow-up.

#### Timeliness and equity

Timeliness and equity are the two remaining quality indicators recommended by the Institute of Medicine [4]. Of the 12 studies, one study assessed timeliness. Preen et al. [66] found general practitioners reported satisfaction with the timely communication resulting from the intervention. Three studies [66, 70, 73] assessed equity and access to services and found that people in their intervention groups were more likely to be referred to community-based services.

### Bias assessment

The potential for selection bias was assessed in terms of the adequacy of random sequence generation and allocation concealment [38]. Random sequence generation was identified as adequate with low risk of selection bias in nine of the 12 studies with three studies providing insufficient information about how the random sequence generation was conducted [66–68]. Allocation concealment was assessed as adequate with low risk of selection bias in eight of the 12 studies with four studies providing inadequate information about how this was undertaken [66, 68–70]. In the Cochrane tool, performance bias is the potential bias resulting from knowledge of research participants and research staff of the interventions that participants were allocated to [38]. No studies were identified as low risk in regard to performance bias.

Detection bias, according to the Cochrane bias assessment tool, is the potential for bias resulting from outcome assessors’ knowledge of the interventions that participants were allocated to [38]. Of the 12 studies, five provided insufficient information to assess the risk of detection bias as these studies did not report if the outcome data collectors were blinded to participant group [66, 68, 70–72]. The study by McInnes [73] was assessed to have low risk of detection bias for service utilisation outcomes but did not report how this risk was managed in relation to questionnaire data.

The risk of attrition bias, the potential for biased conclusions resulting from incomplete outcome data [38], was unclear across most of the included studies with exception to Coleman [69] where this risk was assessed as low.

There was a low risk of reporting bias, bias associated with the selection of particular outcomes for reporting [38], across 11 of the 12 studies with one study providing insufficient information to make an assessment [68]. The potential for other sources of bias was assessed as low risk in three studies [69, 74, 75] and unclear across the remaining nine studies. Findings from the bias assessment of the 12 studies are presented in Table 6.

**Table 6:** **Bias assessment – included studies (n = 12)**

	Risk of bias						
	Selection bias ^1^		Performance bias ^2^	Detection bias ^3^	Attrition bias ^4^	Reporting bias ^5^	Other bias ^6^
First author (year)	Random sequence generation	Allocation concealment	Blinding of participants and personnel	Blinding of outcome assessment	Incomplete outcome data	Selective reporting	Other sources of bias
Coleman (2006) [69]	Low risk	Unclear risk	Unclear risk	Low risk	Low risk	Low risk	Low risk
Enguidanos (2012) [74]	Low risk	Low risk	Unclear risk	Low risk	High risk (self-efficacy, service satisfaction) Low risk (service utilisation)	Low risk	Low risk
Hansen (1995) [70]	Low risk	Unclear risk	High risk (personnel) Unclear risk (participants)	Unclear risk	Unclear risk	Low risk	Unclear risk
Legrain (2011) [71]	Low risk	Low risk	High risk (personnel) Unclear risk (participants)	Unclear risk	Unclear risk	Low risk	Unclear risk
Lim (2003) [76]	Low risk	Low risk	Unclear risk	Low risk	Unclear risk	Low risk	Unclear risk
McInnes (1999) [73]	Low risk	Low risk	Unclear risk	Low risk (service utilisation data) Unclear risk (questionnaire data)	Unclear risk	Low risk	Unclear risk
Naylor (1990) [72]	Low risk	Low risk	High risk (personnel) Unclear risk (participants)	Unclear risk	Unclear risk	Low risk	Unclear risk
Naylor (1994) [68]	Unclear risk	Unclear risk	Unclear risk	Unclear risk	Unclear risk	Unclear risk	Unclear risk
Naylor (1999) [77]	Low risk	Low risk	High risk (personnel) Unclear risk (participants)	Low risk	Unclear risk	Low risk	Unclear risk
Naylor (2004) [75]	Low risk	Low risk	High risk (personnel) Unclear risk (participants)	Low risk	Unclear risk	Low risk	Low risk
Preen (2005) [66]	Unclear risk	Unclear risk	Unclear risk	Unclear risk	Unclear risk	Low risk	Unclear risk
Weinberger (1996) [67]	Unclear risk	Low risk	Unclear risk	Low risk	Unclear risk	Low risk	Unclear risk

^1^Selection bias refers to the adequacy of randomisation processes (random sequence generation) and the adequacy of the concealment of allocation to intervention group (allocation concealment) [38].

^2^Performance bias is the knowledge of research participants and research staff of the interventions that participants were allocated to [38].

^3^Detection bias is outcome assessors’ knowledge of the interventions that participants were allocated to [38].

^4^Attrition bias was the potential for biased conclusions resulting from incomplete outcome data [38].

^5^Reporting bias referred to the selection of particular outcomes for reporting [38].

^6^The potential for other sources of bias (other bias) was also appraised [38].

## Discussion

This review synthesised evidence about the quality of transitional care for older people transitioning from hospital to home in order to produce recommendations for research and practice.

Transitional care interventions examined in the 12 studies were conducted by a range of health and social care professionals, and by older people including advanced practice nurses [68, 72, 74, 75, 77], general practitioners and practice nurses [66, 67, 73], the older person and their carer with support from a transition coach [69], case managers [76] and geriatricians [70, 71]. This indicates that transitional care can be undertaken by a range of health professonal disciplines and importantly, by older people and carers themselves with appropriate support.

Numerous outcomes were assessed [66–77] with mixed findings. Results from the included studies indicate that, except for general practitioner and practice nurse interventions, transitional care delayed and prevented early re-hospitalization. Outcome data in relation to length of stay, costs and quality of life were inconclusive. Notably, a recent Cochrane systematic review by Shepperd et al. [12] found transitional care resulted in cost shifting from the acute to community sector rather than a reduction in costs for the health system as a whole.

Findings indicate that general practitioner and practice nurse interventions were not effective in reducing re-hospitalization rates [67, 73] or length of stay [66, 73]. The study by Weinberger et al. [67] found higher rates of re-hospitalization following their intervention and if re-admitted, the veterans in their study had longer stays in hospital. The veterans who participated in this study also reported low quality of life and may have been in particularly poor health at discharge. It is possible that the transitional care intervention resulted in earlier identification of ill health among these participants with subsequent re-hospitalization [67]. No other included studies targeted veterans and findings from this study may be limited in generalizability to older US veterans. Findings of McInnes et al. [73] and Preen et al. [66] were difficult to interpret because in both studies the intervention was not fully implemented. Only 42% of general practitioners contributed to the discharge plan in the study by Preen et al. [66] and only 52% of patients had general practitioner input into their discharge plan in the study by McInnes et al. [73]. The low rates of participation by general practitioners in both studies highlights the challenges associated with additional work responsibilities in transitional care for primary care providers and suggest that in these two studies the intervention was not feasible for general practitioners. Additionally, the sample size was insufficient to detect an intervention effect in the study by Preen et al. [66].

Effectiveness in terms of symptom management was not specifically studied as an outcome/s in the included studies. This is of concern given findings from two Australian descriptive studies [78, 79] where people reported symptom exacerbation at discharge and an absence of assistance with symptom management and functioning in relation to pain, fatigue, loss of mobility, and grief during care transitions from hospital to home.

Results [66–77] also highlighted the potential for transitional care to result in improved satisfaction for older people however caregiver satisfaction has not been measured. There is also limited understanding of the burden to caregivers.

Timeliness, equity and access are described as part of the intervention in each included study [66–77]. However, consistent with the findings from other research [10, 27] outcomes assessing timeliness, equity and access have not been clearly reported in this research.

Research included in this systematic review [66–77] suggests that measures of re-hospitalization rates or length of stay have been consistently studied in the general transitional care experimental research since 1990, indicating an outcome focus on select quality elements related to effectiveness, safety and efficiency for inpatient services. Other indicators of quality in transitional care, as recommended by the Institute of Medicine [4], Department of Health [8], and Australian Commission on Quality and Safety in Healthcare [6], have not been a consistent outcome focus, suggesting gaps in understanding about timeliness, equity, family/carer centred care and symptom management for older people [10, 27].

Person and family centred care is described as a part of interventions in the included studies [66–77]. Reporting of outcome measures of person centred care has been focussed on patient satisfaction. Six of the twelve identified studies assessed patient satisfaction following the transitional care intervention [66, 67, 73–75, 77]. Only two studies included carer burden [68, 76]. There was little evidence about the 'experience’ of older people and their family/carers although qualitative studies [2, 78, 80, 81] have described problems and unmet needs from older peoples’ perspectives and experiences associated with ineffective transitional care. No studies specifically assessed emotional support for older people and their families/carers.

Although self-management and education were described as components of interventions in the included studies in particular in the intervention by Coleman and associates [64, 69], outcome evidence about self-care and self-management related to older people and their carers’ use of the health care system was limited.

These results are of particular interest because older people and their families/carers are increasingly expected to self-care at home following early discharge and they are expected to navigate complex health care systems [3, 13, 23]. Although Coleman and colleagues [64, 69] conducted focus groups to ascertain what older people wanted in transitional care, no other study contained reports about the involvement of older people and their carers/family in the design of the transitional care intervention that they tested.

In other literature, Naylor [82] and Bauer et al. [10] found that many older people and their family/carers reported unmet discharge needs about information and access to services in the community, and they were not involved in discharge related decisions. Additionally, Bauer et al. [10] found that family carers reported frustration with discharge planning processes, lack of information and poor communication with health practitioners.

### Bias assessment

Findings were mixed in relation to potential sources of bias across the 12 studies. Over 40% of articles did not provide adequate information to accurately assess the risk of bias, suggesting a need for improved reporting about how methods were implemented and about how attrition of participants was managed. Overall, there was low risk of selection bias, however some studies provided insufficient information to assess this risk in relation to the randomisation process [66–68], or how allocation concealment was undertaken [66, 68–70]. No studies were identified to have low risk in regard to performance bias therefore there is potential risk of bias in this regard [38]. Notably, blinding of personnel to group allocation would not be possible for complex health and social care interventions such as transitional care as practitioners conducting the intervention would be aware that they were doing so. No studies reported on the blinding of participants, therefore the risk of performance bias in relation to participants is not known. Of the 12 studies, five provided insufficient information to assess the risk of detection bias [66, 68, 70–72]. The reporting of missing data and how these data were managed was mixed across the 12 studies, indicating unclear risk and potential for attrition bias. There was a low risk of reporting bias across the 12 studies with only one study providing insufficient information to make an assessment [68]. The potential for other sources of bias was assessed as low risk in three studies [69, 74, 75] and unclear across the remaining nine studies.

### Limitations of the current evidence base

In all studies, the transitional care intervention was compared with standard hospital discharge. However, standard hospital discharge was not clearly described and it was therefore not known what the comparison control conditions entailed. All studies included description of interventions in terms of particular quality indicators such as person and family centred care and timeliness, however with exception to patient satisfaction surveys conducted in half of the studies, there was limited reporting of outcome assessment of these quality indicators.

### Limitations of the review

Older people included in the review comprised those aged over 60 years. This potentially includes a wide range in age and a group of people with different health needs. The average ages of people in the studies were specified to provide more focused information. Additionally, the review did not capture grey literature, publically available literature not published in peer review journals; therefore all relevant research may not have been included. Only English language publications were included, therefore the review synthesises the best available evidence published in English only.

## Conclusion

Despite these limitations, findings from this review suggest that there are gaps in the evidence base regarding the quality of transitional care interventions for older people and their families/carers where quality is assessed in terms of effective, efficient, safe and low risk, timely, equitable and person and family centred care. There is a need for improved understanding and evidence about the quality of transitional care for older people and their carers in particular domains of person and family centred care; the patient and carer experience, carer burden and support, and emotional support for older people and their carers during care transitions. This is of particular concern as older people and their families/carers are discharged early and expected to self-care and navigate complex and fragmented systems of care independently. There is a need for improved understanding about outcomes in relation to equity and timeliness in care transitions for older people and their carers. The results from this review highlight that self-management and health outcomes including those assessing symptom management require stronger focus in this literature.

In view of the changing health care context and de-hospitalisation of health and aged care, and because care transitions are increasingly complex, the results also suggest there is a need for research that involves the person and their family/caregiver in the design of high quality transition care interventions in order to meet the needs of older people and their families/carers. The shift in responsibility for health and aged care from acute inpatient settings to the community sector and to family and carers means that older people and their families should be involved in planning and decisions about their care and identifying what would be of most assistance to them.

## Electronic supplementary material

Additional file 1: **Data extraction tool.** (DOC 32 KB)
