# Supplementary material for: Quality care outcomes following transitional care interventions for older people from hospital to home: a systematic review
Source: BMC Health Serv Res. 2014 Aug 15;14:346. doi: 10.1186/1472-6963-14-346 (PMC4147161; doi:10.1186/1472-6963-14-346)
Supplement: Supplementary file 1 — Additional file 1: Data extraction tool. (DOC 32 KB) [file 12913_2014_3453_MOESM1_ESM.doc]

**Additional file 1** Data extraction tool

| Variable | Data |
| --- | --- |
| *Study characteristics* | |
| Authors |  |
| Journal title |  |
| Year of publication |  |
| Country (sample) |  |
| N |  |
| Age |  |
| Design |  |
| Comparison |  |
| *Intervention & outcomes* | |
| Transition settings |  |
| Description of transition care intervention |  |
| Quality outcomes | Re-hospitalization  Length of stay  Costs  Other effectiveness  Other safety & risk  Timeliness  Equity  Person centred care  Family centred care  Other |
